# Resources for oral health in Africa

**DOI:** 10.3389/froh.2025.1540944

**Published:** 2025-02-07

**Authors:** Moréniké Oluwátóyìn Foláyan, Ahmed Bhayat, Sara S. Mikhail, Nicaise Ndembi, Maha El Tantawi

**Affiliations:** ^1^ The Africa Oral Health Network (AFRONE), Alexandria University, Alexandria, Egypt; ^2^ Department of Child Dental Health, Obafemi Awolowo University, Ile Ife, Nigeria; ^3^ Department of Community Dentistry, University of Pretoria, Pretoria, South Africa; ^4^ Department of Pediatric Dentistry and Dental Public Health, Alexandria University, Alexandria, Egypt; ^5^ Division of Epidemiology and Prevention, Institute of Human Virology, University of Maryland School of Medicine, Baltimore, MD, United States

**Keywords:** dental caries, periodontal diseases, lip neoplasms, health workforce, dental technicians, dental assistants, partnerships

## Abstract

Oral health in Africa is often overlooked despite its substantial impact on overall health and well-being. Currently, Africa has a very high prevalence of dental diseases, including untreated dental caries in permanent teeth, severe periodontal disease and oral cancer. Dental human resources are also very low, with dentists ranging from 1.77 to 0.03 per 10,000 population across the continent. The number of technicians also varies across the continent from 0.17 to.0.1 per 10,000 population. Southern Africa has the highest median dental assistants and therapists per 10,000 population ratio (0.2), whereas Northern Africa has no dental assistants or therapists. In addition, limited infrastructure and funding have resulted in significant oral health disparities, leaving large portions of the population without adequate access to oral health services. Only 57% of African countries have developed an oral health policy that sets targets and implementation strategies. African countries have also been shown to spend a fraction of their health budget on oral health care, albeit that dentistry is one of the most expensive medical services. Addressing these gaps requires addressing the oral health workforce needs, facilitating the development of oral health policies built on context-specific evidence, and guiding practice and policy. In addition, partnerships are needed to support innovation, sustainability and monitoring of the instituted oral health programs.

## Introduction

Oral health, essential for basic functions like eating, speaking, and socialising, is often overlooked in African public health policies despite its impact on physical, mental, and social well-being (1). Across the continent, a high burden of untreated oral diseases—from dental caries and periodontal disease to oral cancers, HIV-related conditions and noma—continues to compromise the quality of life of millions. An estimated 43.7% (480 million people) of the population in the World Health Organisation (WHO) Africa Region (AFR) live with poor oral health (2). This lack of focus not only marginalises oral health but also limits the effectiveness of health systems overall.

However, there is growing recognition of the need for an integrated, community-focused approach to oral health. Organisations like the WHO, the International Dental Federation (FDI), and the African Union emphasise oral health as a component of public health. A robust response requires adequate resourcing (3).

The successful integration of oral health into Africa's public health agenda requires a multi-faceted approach that engages healthcare professionals, communities, policymakers, and global health organisations. Resourcing is key, and collaborations with institutions, industry leaders, researchers, civil society, and international organisations are important. By combining the needed resources, Africa can build a resilient oral healthcare system that provides quality evidence-based services and formulates policies.

This manuscript explores the challenges facing oral health in Africa and proposes a collaborative framework for strengthening oral health systems in Africa. It examines the potential roles of various stakeholders in addressing current gaps and highlights strategies to harness resources effectively. Building a resilient oral healthcare system in Africa requires a multi-faceted approach that acknowledges local expertise, enhances community engagement, and supports evidence-based policy development, positioning oral health as a critical priority within Africa's public health agenda.

## Prevalent oral health conditions in Africa

The most common oral diseases in Africa are heavily influenced by factors such as limited access to oral healthcare, poor oral hygiene, inadequate public health education, and systemic socioeconomic challenges. One of these is dental caries. Dental caries is highly prevalent among children and adults in Africa. Studies indicate that dental caries affect deciduous and permanent teeth across various populations. In 2019, 28.5% of the population in the WHO African Region had untreated caries of permanent teeth, and 38.6% of children aged 1–9 years had untreated caries of deciduous teeth (4). In addition, up to 36% of children aged 12 in Africa have dental caries (5). Furthermore, 2.8% of persons aged 15 years suffered from severe periodontal disease, and between 0.4 and 6.6 per 100,000 people had cancers of the lip and oral cavity in 2020 (4).

The burden of prevalent oral diseases in Africa is high, as seen in Table 1. The data was extracted from the 2022 Global Oral Health Status Report (6). We divided the African countries into five regions following the African Union classification: Northern, Southern, Eastern, Western, and Central Africa (7). The table presents the burden of three major oral diseases: untreated caries in permanent teeth, severe periodontal disease, and lip and oral cavity cancer (6). We calculated the mean (SD) or median (interquartile range) of the countries for each region using the values reported in the Global Oral Health Status Report (6).

**Table 1:** Burden of major oral diseases in Africa.

Regions	Prevalence of untreated caries of permanent teeth in people 5+ years (%) Mean (SD)	Prevalence of severe periodontal disease in people 15+ years (%) Mean (SD)	Lip and oral cavity cancer (Number of new cases) Median (IQR)
Northern Africa (*n* = 7)	33.72 (2.88)	18.4 (5.07)	269.5 (58.25, 878)
Central Africa (*n* = 9)	29.48 (1.49)	24.47 (3.52)	71 (20.75, 200.75)
Southern Africa (*n* = 10)	31.47 (3.86)	22.53 (2.4)	116.5 (52.25, 316.25)
Eastern Africa (*n* = 14)	30.54 (4.23)	18.71 (4.45)	175 (52, 522.5)
Western Africa (*n* = 15)	30.29 (3.51)	26.75 (4.5)	74 (36, 181)

SD, standard deviation; IQR, Inter-quartile ratio.

The prevalence of untreated caries in permanent teeth for people aged 5+ years is highest in Northern Africa (33.72%; SD: 2.88), followed by Southern Africa (31.47%; SD: 3.86), and lowest in Central Africa (29.48%; SD: 1.49). The overall prevalence of untreated caries in permanent teeth across the continent suggests this condition is widespread.

In addition, the prevalence of severe periodontal disease in people aged 15+ years is highest in Western Africa (26.75%; SD: 4.5), followed by Central Africa (24.47%; SD: 3.52), and lowest in Eastern Africa (18.71%; SD: 4.45). On the other hand, Northern Africa experiences the highest number of new cases of lip and oral cavity cancer (median: 269.5; IQR: 58.25, 878), followed by Eastern Africa (median: 175; IQR: 52, 522.5), whereas Western Africa (median: 74 cases; IQR = 36, 181) and Central Africa (median: 71 cases; IQR: 20.75, 200.75) report fewer new cases.

The data suggests that the burden of oral diseases in Africa varies across regions. This may be due to complex socioeconomic, dietary, and healthcare-related factors. For example, Northern Africa shows a high prevalence of untreated caries, plausibly due to increased access to sugar-rich diets advancing healthcare infrastructure but limited access to preventive dental care as healthcare access does not match the transiting economies (8). On the other hand, Southern Africa has moderate caries rates, reflecting mixed socioeconomic conditions (9), while Western and Central Africa face higher periodontal disease prevalence (10), likely linked to poor oral hygiene and limited dental care access (11). Lower lip and oral cavity cancer rates in these regions may reflect underdiagnosis due to inadequate screening (12). Disparities in data quality and reporting may lead to underestimations in some regions. Cultural practices, environmental exposures (tobacco, alcohol, ultra violet light), and demographic factors, including older age distributions, further shape disease patterns (13).

These and several other factors contribute to Africa's neglect of oral diseases. The subsequent sections discuss further why these factors must be addressed to ensure oral health response in Africa is effectively resourced.

## Oral health workforce

### Shortage

Currently, Africa faces a severe health crisis, carrying 24% of the global disease burden with only 3% of the world's health workforce (14). There is a dire shortage of an oral health workforce in Africa, with many African countries having fewer than one dentist per 10,000 people (15, 16), resulting in overcrowded urban clinics and limited access to care in rural areas (17). Overcrowding results from fewer human resources, which leads to fewer dental facilities, which are overburdened. It also results in the maldistribution of human resources as the service providers tend to reside in urbanised communities, leading to a shortage and overcrowding in rural and underserved communities. The shortage of the dental workforce is a critical concern. While the African oral health workforce grew by 311% from 2013 to 2022—The density of dentists per 10,000 population rose from 0.15 in 2013 to 0.37 in 2022, and when dental assistants and therapists are included, the total oral health workforce grew by 283%, increasing from 14,817 in 2013 to 56,772 in 2022. Thus, the significant shortages remain (18). In 2022, 275,893 oral health professionals were needed to meet 70% of UHC targets. However, only 56,772 were available, leaving a gap of 219,121 professionals, with countries like Nigeria, Ethiopia, and the Democratic Republic of Congo facing the largest deficits (19).

Table 2 outlines the distribution of the oral health workforce across the five African regions, highlighting significant disparities in the availability of oral healthcare professionals per 10,000 population. The most recent data were extracted from the WHO Global Health Observatory (20–22). The median and interquartile ranges for countries per region were calculated and are shown in the table.

**Table 2:** Oral health workforce distribution across African regions (per 10,000 population).

Regions	Dentists Median (IQR)	Dental prosthetic technicians Median (IQR)	Dental Assistants and Therapists Median (IQR)
Northern Africa (*n* = 7)	1.77 (1.1, 4.98)	0.17 (0.08,.)	0 (0, 0)
Central Africa (*n* = 9)	0.03 (0.01, 0.16)	0.01 (0,.)	0.1 (0,.)
Southern Africa (*n* = 10)	0.16 (0.09, 0.45)	0.08 (0.03, 0.15)	0.2 (0.1, 0.5)
Eastern Africa (*n* = 14)	0.18 (0.06, 1.27)	0.04 (0, 0.11)	0.1 (0, 0.2)
Western Africa (*n* = 15)	0.03 (0.02, 0.15)	0.02 (0.01, 0.06)	0 (0, 0.1)

SD, standard deviation; IQR, Inter-quartile ratio.

Northern Africa has the highest median number of dentists per 10,000 population (1.77), followed by Eastern Africa (0.18) and Southern Africa (0.16). Central and Western Africa had the lowest dentist to 10,000 population ratio (0.03 each). For dental prosthetic technicians, Northern Africa leads with a median of 0.17 technicians per 10,000 population, while Western Africa and Central Africa had very low median technicians to 10,000 population ratios (0.02 and 0.01, respectively). Southern Africa has the highest median dental assistants and therapists per 10,000 population ratio (0.2), whereas there were no dental assistants or therapists in Northern Africa [median (IQR): 0 (0,0)]. These disparities underscore the critical need for targeted interventions to address workforce shortages, especially in regions with limited dental care, to improve oral health outcomes across the continent.

### Training needs

The needs of each country must be identified, and the necessary training must be carried out. A needs assessment based on the population and the number of people in the oral health workforce can determine the needs, and based on the disease burdens, the type of care can also be identified. Academic dental institutions and research centres are pivotal in identifying the continent and countries' unique oral health challenges (23). Dental schools and private stakeholders must invest in training so that an adequate supply of oral health workforce can be established. They can generate evidence to inform locally relevant oral health policies, build partnerships, and expand research focus to the whole continent (24). Another factor is the emigration of the oral health workforce to developed countries. This movement exacerbates the shortage and must be addressed nationally and continentally.

National dental associations can build capacity and promote resources. These professional associations can drive capacity-building initiatives and strengthen oral healthcare services through training and leveraging their global networks through organisations like the FDI to bring innovative solutions, technological advancements, and best practices to the African oral health systems.

The “African Summit” in Cape Town marked a critical step forward in addressing Africa's oral health challenges through a unified, collaborative approach. Attended by presidents from 16 African National Dental Associations, FDI stakeholders, WHO representatives, and government delegates, the summit produced a declaration setting three core priorities for the African strategy: strengthening the credibility of national dental associations, fostering leadership and management skills, and promoting effective peer-to-peer information exchange. These principles support sustainable growth and collaboration in African oral health development (25).

## Oral health policies

Policy gaps play a significant role in Africa's marginalisation of oral health. Only 57% (26 out of 45) of African countries have a national oral health policy (26). The lack of a policy exacerbates the weakening oral health sector as it creates fragmented care, a reduced allocation of funds for oral health, a lack of emphasis on curative rather than preventive care, minimal focus on preventive programs, and limited education about oral hygiene. Even in countries with a policy, it is often not prioritised and applicable. The minimal integration of oral healthcare services into healthcare undermines the ability to reach a larger population segment through the public healthcare system (3). It is estimated that there will be approximately 98,745 public healthcare facilities in 50 countries in the AFR by the end of 2022 (27). Primary healthcare (PHC) centres are the most numerous, offering essential services like immunisation, maternal health care, and basic outpatient care, potentially reaching 80% of the population, as Ethiopia achieved in 2014 (28). The secondary healthcare facilities include district hospitals and some specialised clinics. The tertiary healthcare centres are major hospitals, which are fewer in number and are often located in large cities, managing complex health conditions that demand specialised expertise and targeted treatment. Only 5%–10% of the population need tertiary care services (29). Due to the poor integration of oral health care into PHC, many oral PHC services are provided at tertiary healthcare centres (30). Access to oral health care then becomes limited.

Also, public healthcare in Africa is underfunded (31) and focused on infectious diseases like HIV/AIDS, Ebola, and mpox, drawing resources and attention away from areas like oral health. Limited financial resources are allocated to oral health, with about 70% of countries in the AFR spending less than US$ 1 per person per year on oral care (32). In addition, the high cost of dental care and out-of-pocket expenses prevent many from seeking timely treatment (33).

Strengthening national dental associations is crucial for influencing oral health policy in Africa. Developing leadership and management skills in dental professionals addresses gaps in health systems, empowering them to lead policy efforts and drive organisational growth. This leadership is vital for prioritising oral health in national health planning. Promoting peer-to-peer information exchange enhances regional collaboration, enabling countries to share best practices and address service delivery gaps.

## Context-specific evidence to guide practice and policy

There is a gap between research and clinical practice in Africa, with over-reliance on imported science produced in different settings to address different problems. Evidence-based solutions should be generated for the local oral health problems. However, the capacity to meet this need varies across countries and dental academic institutions (34). There is generally modest competency to conduct cutting-edge oral health research in the continent (35).

African academic dental institutions and research centres are pivotal in understanding the continent's unique oral health challenges (2). Dental schools are key stakeholders in Africa's oral health resourcing ecosystem because they are uniquely positioned to partner with others due to their expertise, resources, and mission. They can generate evidence to inform locally relevant oral health policies, build partnerships, and expand research focus to the whole continent (2).

Key factors hindering the progress in research-based evidence generation for practice and policy are the uninspiring biomedical curricula that fail to encourage research as a career choice (36), lower remuneration for researchers than their clinical counterparts (37), and the pressure for immediate employment to support families, which deters medical and dental graduates from pursuing research careers (38). In addition, challenges with grant writing (37, 39), along with weak research networks, reduce the retention of scientists in the continent. Many countries allocate 0.1%–0.5% of their gross domestic product (GDP) to science and technology—far below UNESCO's recommended minimum of 1% GDP (40, 41) with even more limited funding for dental research.

Postgraduate dental programs could support translational oral health research relevant to Africa's populations and cultivate leaders in oral health to drive inclusive health policies (42). In addition, limited mentorship opportunities also contribute to poor research involvement in Africa (43). Thus, programs which support early career researchers are important to build a pipeline of skilled professionals committed to advancing oral health in Africa. Structured mentorship programmes and training for researchers on the continent for six months have increased research activity, confidence and understanding of research and fostered a positive research culture (44). Peer research support can address mentorship gaps in academic institutions (45). Distance learning expands health research training, and e-mentorship helps overcome barriers like distance and time constraints (44).

Initiatives such as the Africa Oral Health Network (46), the African Oral Pathology Research Consortium (47), the African Craniofacial Anomalies Network (48), and Africa Unite for Paediatric Dental Oral Health Research are enhancing mentorship and research collaboration across the continent. These programs foster south-south collaboration among African researchers, addressing the historical gap in research partnerships, where most collaborations occur with partners from the global North and the former colonising countries (46). Academic institutions can also connect with global research networks to fund innovative oral health research and contribute meaningfully to global discourse. One such effort is the Joint International Master of Oral Public Health (JIM-OPH) program, a two-year competency-based Erasmus Mundus programme developed by collaboration among European and African public health researchers and funded by the European Education and Culture Executive Agency. The curriculum builds dental public health and research skills to promote oral health in Africa and beyond (49).

## Partnership to support innovation, sustainability and monitoring

The private sector, including pharmaceutical and dental equipment manufacturers, holds untapped potential to bridge the resource gaps needed for oral health in Africa. Engaging the private sector allows expanding access to care, prioritising underserved populations, promoting prevention, addressing healthcare professionals' shortage, and engaging policymakers through innovation and tailored solutions (50). Industry can support research, provide affordable products, and contribute to training healthcare workers to use new technologies. The private sector can collaborate with African universities by investing in research and development focusing on affordable oral health solutions for low-resource settings and improving access to essential products, such as fluoride toothpaste and topical fluorides, particularly in rural areas. A corporate social responsibility agenda supporting oral health can scale up oral health programs in collaboration with African governments by integrating oral health into the broader public health and sustainability efforts (51).

More partnerships with the medical sectors are required to prioritise oral health and implement oral health interventions using a common risk factor approach. This will extend to a larger audience and will not be as resource-intensive as the same programs being done in isolation. Oral health education and interventions can also be implemented at Primary Health Care facilities and through community workers who often have a larger footprint in the communities than the dental sector alone.

Another important partnership is between the oral health community and civil society organisations (CSOs). CSOs bring grassroots insights and credibility to oral health initiatives, especially in underserved and rural communities. They can advocate for policies prioritising oral health by pushing governments to increase funding and support evidence-based policies (52). They can also foster partnerships between researchers, governments, and the private sector as neutral intermediaries, creating innovative solutions and pooling resources to tackle relevant issues (53). In addition, CSOs can play a key role in community-led monitoring by gathering data directly from communities to bring attention to urgent oral health needs, monitoring service quality, and holding governments accountable (54). Community-led monitoring empowers communities to voice their health needs, providing real-time data on service gaps, access issues, and specific oral health risks, strengthening advocacy for policy changes and resource allocation in underserved areas (55). Partnering with local clinics, schools, and dental practitioners, CSOs can promote community-centred design to align national health policies with local oral health needs.

## Discussion

Oral health resources encompass a diverse ecosystem where each stakeholder is vital in building an inclusive, sustainable, and accessible oral health landscape. African academic institutions drive impactful research, train future professionals, and advocate for comprehensive health policies. Pharmaceutical and dental technology companies support by funding research, expanding access to affordable care products, and backing community reach. Researchers affiliated with African institutions generate local evidence on oral disease cultural practices, contextualising solutions and advocating for tailored policies. Civil society organisations champion oral health as a fundamental right, promote government accountability, and support communities in designing and monitoring the services they receive. Finally, policy actors, including ministries of health and the Africa Centers for Disease Control and Prevention, establish priorities and integrate oral health into broader health agendas. This collaborative ecosystem can enhance sustainable oral health outcomes through shared knowledge, policy alignment, service delivery, and advocacy.

Several critical areas highlighted demand immediate attention and intervention to create a collaborative ecosystem that can deliver sustainable oral health outcomes. The government need to do more to curb the emigration of trained professionals. About 70,000 skilled professionals are estimated to emigrate from Africa annually (56), which has implications for the annual flow into the oral health job market. Strategies include adopting evidence-based, context-specific oral health policy frameworks, improving working conditions, offering competitive salaries, and creating opportunities for professional development. Integrating oral health services into PHC systems, allocating healthcare budgets for oral health services, and expanding the scope of public dental healthcare delivery to include preventive services can improve oral health infrastructure development, workforce expansion, and public health initiatives.

In addition, academic institutions need to be more active, directly and purposefully engaged with achieving national goals. This calls for increased research funding and the building of research capacity across African countries. This can be achieved by supporting not only postgraduate programmes but also implementing structured mentorship opportunities, early-career researcher training, strengthening regional research networks, and fostering South-South collaboration, critical actions needed to empower African researchers to develop solutions tailored to local challenges. Multi-sectoral partnerships, particularly with the broader healthcare sector and the private, are also crucial to drive these strategies and stimulate innovation in dental technologies and mobile health solutions, particularly in remote areas.

Importantly, advocacy and awareness efforts are critical to promoting the importance of oral health in Africa. National and regional campaigns must be launched to educate the public about the links between oral health and overall well-being and advocate for including oral health in national health policies and budgets. Policymakers should be targeted with evidence-based presentations highlighting the social and economic consequences of oral health neglect, focusing on the long-term benefits of investing in oral health. Evidence can be further strengthened through monitoring and evaluation mechanisms that track the progress of oral health initiatives and ensure accountability. The regional oral health observatory offices in Africa must be strengthened to better monitor oral disease trends, assess the effectiveness of policies and interventions, and provide a comprehensive overview of the continent's oral health status. Standardised metrics aligned with the Sustainable Development Goals (SDGs) should be adopted to assess oral health outcomes, and annual reports should be published to guide evidence-based decision-making and resource allocation. These efforts will contribute to better oral health outcomes and support the broader goal of achieving universal health coverage (UHC) across the continent.

## Conclusion

This paper explores the challenges facing oral health in Africa and highlights the variations in the African continent regarding the dental disease profile, oral health workforce and availability and utilisation of a national oral health policy. It also identified a lack of potential roles of various stakeholders in addressing the current gaps. It provided suggestions on how countries could build a more resilient oral healthcare system in Africa that acknowledges local expertise, enhances community engagement, and supports evidence-based policy development, positioning oral health as a critical priority within Africa's public health agenda. With the support of allies, national dental associations, and global partners, Africa has the potential to transform its oral health landscape, creating a future where oral health is an integral part of overall health and accessible to all. Improving oral health outcomes in Africa requires a coordinated effort that maximises existing resources, strengthens partnerships, and builds capacity across all levels of society. By bringing together institutions, industry, researchers, civil society, and international associations, Africa can develop a sustainable oral health framework that addresses current disparities and promotes long-term health equity to improve oral health.

## Data Availability

Publicly available datasets were analyzed in this study. This data can be found here: Not applicable.
